# Enhanced quantal release of excitatory transmitter in anterior cingulate cortex of adult mice with chronic pain 

**DOI:** 10.1186/1744-8069-5-4

**Published:** 2009-01-27

**Authors:** Hiroki Toyoda, Ming-Gao Zhao, Min Zhuo

**Affiliations:** 1 Department of Physiology, Faculty of Medicine, Centre for the Study of Pain, University of Toronto, Toronto, Ontario, Canada; 2 Department of Neuroscience and Oral Physiology, Osaka University Graduate School of Dentistry, Suita, Japan; 3 Department of Pharmacology, Fourth Military Medical University, Xi'an, PR China

## Abstract

The anterior cingulate cortex (ACC) is a forebrain structure that plays important roles in emotion, learning, memory and persistent pain. Our previous studies have demonstrated that the enhancement of excitatory synaptic transmission was induced by peripheral inflammation and nerve injury in ACC synapses. However, little information is available on their presynaptic mechanisms, since the source of the enhanced synaptic transmission could include the enhanced probability of neurotransmitter release at existing release sites and/or increases in the number of available vesicles. The present study aims to perform quantal analysis of excitatory synapses in the ACC with chronic pain to examine the source of these increases. The quantal analysis revealed that both probability of transmitter release and number of available vesicles were increased in a mouse model of peripheral inflammation, whereas only probability of transmitter release but not number of available vesicles was enhanced in a mouse model of neuropathic pain. In addition, we compared the miniature excitatory postsynaptic potentials (mEPSCs) in ACC synapses with those in other pain-related brain areas such as the amygdala and spinal cord. Interestingly, the rate and amplitude of mEPSCs in ACC synapses were significantly lower than those in the amygdala and spinal cord. Our studies provide strong evidences that chronic inflammatory pain increases both probability of transmitter release and number of available vesicles, whereas neuropathic pain increases only probability of transmitter release in the ACC synapses.

## Background

The ACC is involved in major brain functions including learning, memory, and persist pain [1-9]. Recently, a number of studies consistently suggest that the ACC plays important roles in processing pain-related information in humans and in the behavioral responses to noxious stimuli or tissue injury in animals [3,6,10-14]. As for the mechanisms of pain transmission and modulation, it has been proposed that the enhancement of synaptic transmission contributes to chronic pain. For example, we have shown that excitatory synaptic transmission was enhanced in the ACC of mice with persistent inflammatory pain [15] and neuropathic pain [7]. There are at least two main evidences supporting for presynaptic changes of glutamate release in the ACC after the nerve injury: First, we demonstrated that paired-pulse facilitation (PPF), a phenomenon in which activation of a synapse at shorter intervals results in a presynaptic facilitation of transmitter release in response to the second stimulus [16] was apparently reduced in ACC synapses with chronic pain [7,15]. Second, we showed that the rate of MK-801 (NMDA receptor antagonist) blocking was significantly faster in ACC synapses with chronic pain together with the increase of mEPSCs rate [7,15]. Therefore, it is likely that peripheral inflammation or nerve injury produces increases in mean quantal content in ACC synapses.

The quantal content of transmission at a synapse is determined by the number of releasable quanta (*N*) that corresponds to the number of functional vesicles, and the probability (*p*) of each quantum to be released. Although our previous studies demonstrated that presynaptic neurotransmitter release was enhanced by peripheral inflammation and neuropathic pain in ACC synapses [7,15], the exact changes in these quantal parameters remains to be investigated. To examine the source of the enhanced presynaptic neurotransmitter release, here we performed quantal analysis of excitatory synaptic transmission with inflammatory pain and neuropathic pain.

mEPSCs that are observed in the absence of presynaptic action potentials, are frequently used as a parameter to reflect altered synaptic transmission responsible for inflammatory pain [15], neuropathic pain [7] and diabetic neuropathy [17]. The amplitude and decay time constant of mEPSCs are determined by various factors such as the glutamate concentration in the synaptic cleft and the receptor properties; the glutamate concentration in the synaptic cleft is determined by the kinetics of glutamate release, diffusion, binding by the receptors, uptake by the transporters, and synaptic geometry, while the receptor properties are determined by kinetics, density, and spatial distribution [18]. We also compared the characteristics of mEPSCs in pain-related brain regions such as ACC, basolateral amygdala, and spinal cord of adult mice and discuss how such characteristics of mEPSCs maybe related to pain transmission and modulation.

## Results

### Quantal analysis of excitatory synaptic transmission in ACC synapses following CFA injection and nerve ligation

**Figure 1:**
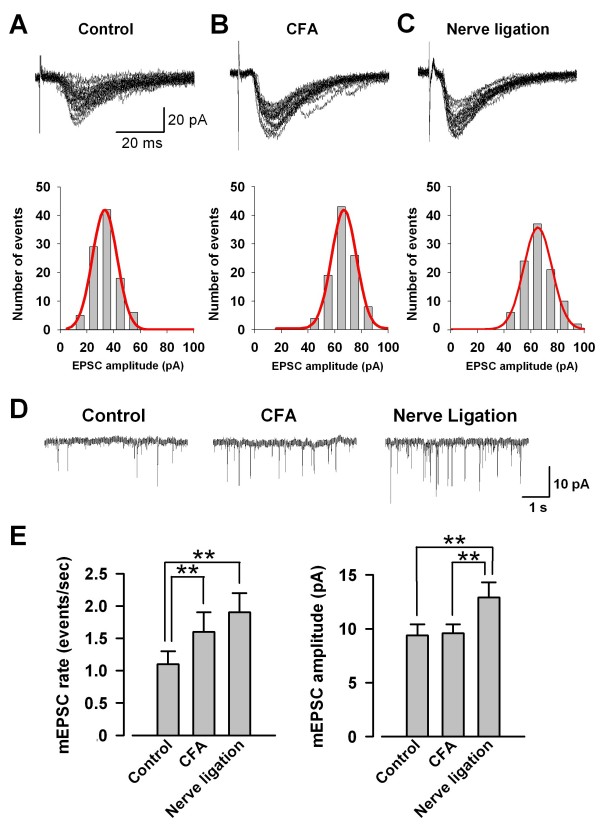
**uEPSCs and mEPSCs in ACC synapses after peripheral inflammation and nerve ligation**. A-C, Traces indicate 20 consecutive uEPSCs recorded at the stimulus intensity of 5 V in ACC neurons from control mouse (A), mouse following CFA injection (B), and mouse with nerve ligation (C), respectively. Representative histogram of peak amplitude of uEPSCs (100 trials) in ACC neurons from control mouse (A), mouse following CFA injection (B), and mouse with nerve ligation (C), respectively, with a Gaussian curve (Bottom). D, Representative mEPSCs recorded in pyramidal neurons at a holding potential of -70 mV from control mice, mice following CFA injection, and mice with nerve ligation. E, The mean rate (left) and amplitude (right) of mEPSCs in neurons from control mice (n = 12), mice following CFA injection (n = 8), and mice with nerve ligation (n = 9). **p < 0.01.

We performed conventional whole-cell patch-clamp recordings from visually identified pyramidal neurons in the layer II/III of ACC slices in mice receiving CFA and in mice with peripheral nerve ligation. First, we compared the unitary EPSCs (uEPSCs) among ACC neurons from control mice, mice following CFA injection, and mice with nerve ligation. The uEPSCs were obtained by delivering focal electrical stimulation to the layer V. As shown in Fig. 1A, uEPSCs in control mice were obtained at the stimulus intensity of 5 V, and the mean amplitude of uEPSCs was 25.7 ± 8.9 pA (n = 12). Subsequently, the same intensity of stimulus was used to record uEPSCs in mice following CFA injection and mice with nerve ligation. We found that uEPSCs were potentiated in mice after CFA injection or peripheral nerve ligation, and the mean amplitude of uEPSCs in mice following CFA injection and mice with nerve ligation were 50.7 ± 8.6 (n = 8) and 52.4 ± 9.6 (n = 9) pA, respectively (Fig. 1B and 1C). After recording uEPSCs, we perfused TTX (1 μM) to obtain mEPSCs that reflect the release of single quanta of neurotransmitter (Fig. 1D). The rates of mEPSCs in mice following CFA injection and mice with nerve ligation were significantly larger (*p *< 0.01, respectively) than that in control mice (control, 1.1 ± 0.2 Hz, n = 12; CFA, 1.6 ± 0.3 Hz, n = 8; Nerve ligation, 1.9 ± 0.3 Hz, n = 9, Fig. 1E). There was no difference in the amplitude of mEPSCs between control mice and mice with CFA injection, while that in mice with nerve ligation was significantly larger (*p *< 0.01, respectively) than those in control mice and mice following CFA injection (control, 9.4 ± 1.0 pA, n = 12; CFA, 9.6 ± 0.8 pA, n = 8; Nerve ligation, 12.9 ± 1.4 pA, n = 9, Fig. 1E). These results indicate that enhanced excitatory synaptic transmission after CFA injection is mainly attributable to an increase in probability of presynaptic neurotransmitter release, whereas that after nerve ligation is caused by not only an increase in probability of presynaptic neurotransmitter release but also an increase of postsynaptic responsiveness.

**Figure 2:**
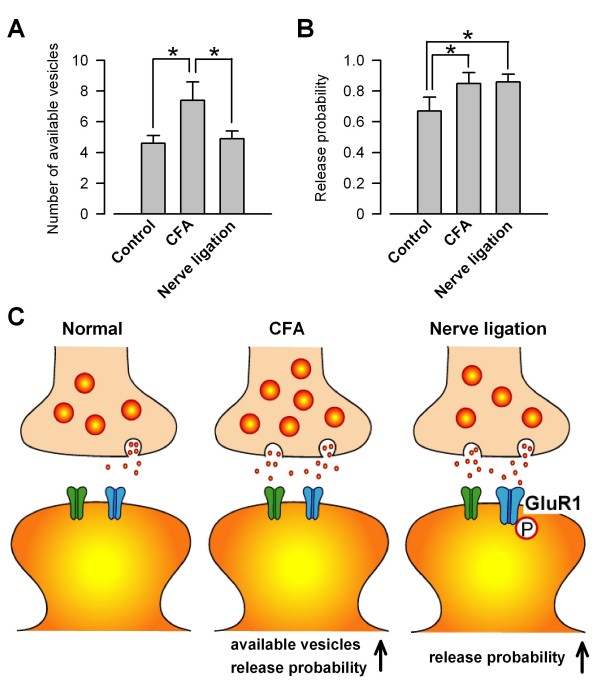
**Number of available vesicles and release probability after peripheral inflammation and nerve ligation**. A and B, Number of available vesicles (A) and release probability (B) in control mice (n = 12), mice following CFA injection (n = 8), and mice with nerve ligation (n = 9). *p < 0.05. C, A model for enhanced quantal release in ACC synapses with chronic pain. In mice following CFA injection, both probability of transmitter release and number of available vesicles are increased in ACC synapses. In mice with nerve ligation, only probability of neurotransmitter release but not number of available vesicles increased in ACC synapses. Upregulated phosphorylation of GluR1 contributes to an increase of postsynaptic responsiveness in mice with nerve ligation.

To evaluate the number of available vesicles and release probability, quantal analysis was performed by using a simple binomial statistics. CV (ratio of SD (σ) to the mean uEPSCs amplitude (μ)) varies with quantal content and reflects changes in presynaptic function including the number of available vesicles (*N*) and release probability (*p*) [19,20]. If we assume binominal statistics, CV and σ^2^/μ (ratio of the variance (σ^2^) to the mean uEPSCs amplitude (μ)) can be expressed as follows:

$$\text{CV}=\sqrt{\frac{1}{N}(\frac{1}{p}-1)},$$

$$\frac{\sigma^{2}}{\mu }=q(1-p),$$

where *N *is the number of available vesicles, *p *is the release probability and *q *is the mean mEPSC amplitude. The number of available vesicles in CFA-treated mice was significantly increased (*p *< 0.05, respectively) in comparison with those in control mice and mice with nerve ligation, although there was no difference between control mice and mice with nerve ligation (control, 4.6 ± 0.5, n = 12; CFA, 7.4 ± 1.2, n = 8; Nerve ligation, 4.9 ± 0.5, n = 9, Fig. 2A). The release probabilities in mice following CFA injection and mice with nerve ligation were also significantly increased (*p *< 0.05 and *p *< 0.05, respectively) in comparison with that in control mice, while there was no difference between those in mice following CFA injection and mice with nerve ligation (control, 0.67 ± 0.09, n = 12; CFA, 0.85 ± 0.07, n = 8; Nerve ligation, 0.90 ± 0.05, n = 9, Fig. 2B). These findings suggest that both the number of available vesicles and the release probability could be enhanced by inflammatory pain, whereas only the release probability could be enhanced by nerve ligation (Fig. 2C).

### Comparison of mEPSCs in ACC, basolateral amygdala, and spinal cord

**Figure 3:**
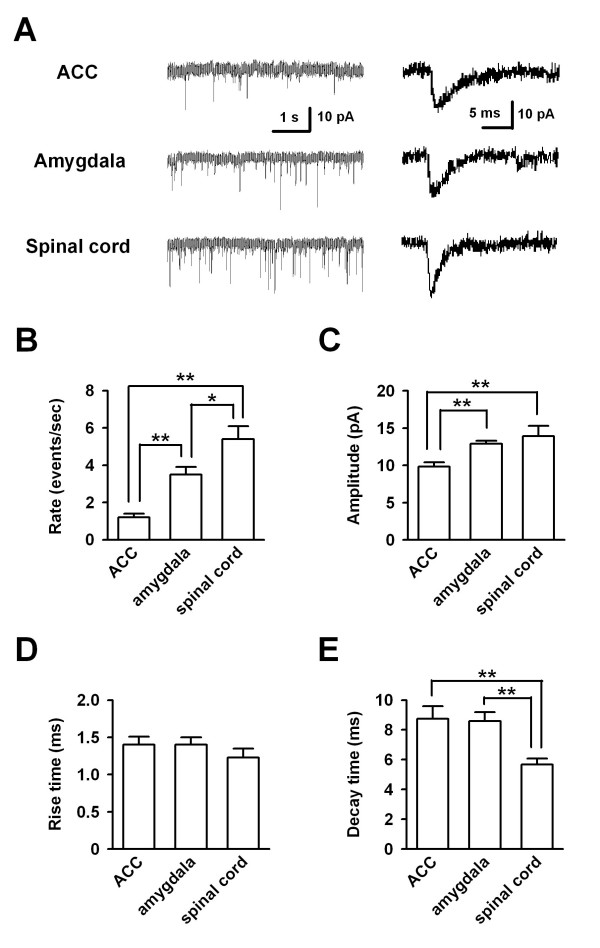
**Characteristics of mEPSCs in ACC, basolateral amygdala, and spinal cord**. A, Representative mEPSCs recorded at a holding potential of -70 mV from ACC, basolateral amygdala, and spinal cord neurons, respectively (left). Individual mEPSCs obtained from respective recordings (right). B-E, Bar graphs showing the averaged rate (B), amplitude (C), rise time (D), and decay time (E) in ACC (n = 12), basolateral amygdala (n = 14), and spinal cord neurons (n = 12), respectively. Note that the rate and amplitude of mEPSCs from ACC neurons are significantly lower than those from amygdala and spinal cord neurons. Also note that the decay time of mEPSCs from spinal cord neurons is significantly smaller than those from ACC and amygdala neurons. *p < 0.05, **p < 0.01.

Next, we recorded mEPSCs from pyramidal cells of ACC and basolateral amygdala and lamina II cells of spinal cord in normal condition (Fig. 3A). In the present study, we decided to record from basolateral amygdala and spinal cord among the pain-related brain regions, since basolateral amygdala is a major region that is known to contribute to pain-related fear/anxiety [21] and superficial lamina of the spinal dorsal horn is the first site of synaptic integration in the pain pathway [22]. First, we compared the rate of mEPSCs among ACC, basolateral amygdala, and spinal cord. Interestingly, the rate of mEPSCs in ACC was significantly lower than those in basolateral amygdala and spinal cord (*p *< 0.01 and *p *< 0.01, respectively), and the rate in basolateral amygdala was also significantly lower than that in spinal cord (*p *< 0.05) (ACC, 1.2 ± 0.2 Hz, n = 12; basolateral amygdala, 3.5 ± 0.4 Hz, n = 14; spinal cord, 5.4 ± 0.7 Hz, n = 12, Fig. 3B).

We also compared the amplitude of mEPSCs among three groups. The amplitude of mEPSCs in ACC was significantly smaller than those in basolateral amygdala and spinal cord (*p *< 0.01 and *p *< 0.01, respectively), although there was no difference between basolateral amygdala and spinal cord (ACC, 9.8 ± 0.6 pA, n = 12; basolateral amygdala, 12.9 ± 0.4 pA, n = 14; spinal cord, 13.9 ± 1.4 pA, n = 12, Fig. 3C). Next, we compared the rise time of mEPSCs and found that there were no differences among three groups (ACC, 1.4 ± 0.1 ms, n = 12; basolateral amygdala, 1.4 ± 0.1 ms, n = 14; spinal cord, 1.2 ± 0.1 ms, n = 12, Fig. 3D). Finally, we compared the decay time of mEPSCs among three groups. The decay time of mEPSCs in ACC and basolateral amygdala were significantly larger than that in spinal cord (*p *< 0.01 and *p *< 0.01, respectively), although there was no difference between ACC and basolateral amygdala (ACC, 8.8 ± 0.8 ms, n = 12; basolateral amygdala, 8.6 ± 0.6 ms, n = 14; spinal cord, 5.7 ± 0.4 ms, n = 12, Fig. 3E). These results suggest that the rate and amplitude of glutamatergic mEPSCs in ACC synapses are lower than other pain-related areas at resting condition.

## Discussion

In the present study, we performed quantal analysis of excitatory synaptic transmission after CFA injection and nerve ligation. This is the first study, to our knowledge, to perform quantal analysis in the animal model of nerve injury. We demonstrate that both the probability of transmitter release and number of available vesicles were increased in mice following CFA injection, while only the probability of transmitter release but not the number of available vesicles was increased in mice with nerve ligation. Thus, it is strongly suggested that presynaptic mechanisms for enhanced excitatory synaptic transmission within the ACC may not be the same as comparing the inflammatory pain model with neuropathic pain model, although both models showed the enhancement of the presynaptic neurotransmitter release [7,15].

Our current study provided the evidences for enhanced transmitter release by quantal analysis. However, we have to keep in mind that synaptic failures observed in this study would be smaller than expected, since we used the stimulus intensity just above the level that gave mostly failures. In general, when focal extracellular stimulation is used to obtain uEPSCs, the stimulus intensity is adjusted to value that demonstrates an all or none behavior of the EPSCs. Therefore, future studies would be necessary to precisely evaluate the probability of transmitter release and number of available vesicles.

### Quantal analysis in the ACC of mice with inflammatory pain and nerve ligation

In mice following CFA injection, the amplitude of eEPSCs and the rate of mEPSCs were potentiated, whereas that of mEPSCs remained unchanged, compared with the results obtained from control mice. As demonstrated by quantal analysis, these observations can be explained by the enhancement of the number of available vesicles together with the increase in the probability of transmitter release. On the other hand, the enhancement of the amplitude of eEPSCs and mEPSCs together with the increase in mEPSC rate were observed in mice with nerve ligation, in comparison with the results obtained from control mice. These observations would be explained by the enhancement of the probability of transmitter release without changing the number of available vesicles and an increase in postsynaptic responsiveness. Taken together, these results indicate that the mechanisms of the enhanced synaptic transmission vary depending on the nerve injury models. Furthermore, these results would partly reflect the locus of synaptic alterations after peripheral nerve injury. For instance, in mice with inflammatory pain, we have previously shown that the enhanced synaptic transmission results from the increased probability of presynaptic neurotransmitter release rather than a possible postsynaptic modification of functional AMPA receptors [15], although we cannot completely rule out the possible involvement of postsynaptic mechanisms due to the enhancement of NR2B-containing receptors [14]. In mice with nerve ligation, the enhanced synaptic transmission was mediated by presynaptic enhancement of glutamate release probability as well as postsynaptic enhancement of AMPA receptor-mediated responses because of the phosphorylation of GluR1 subunit [7]. Increases in mEPSC amplitude after nerve ligation could also reflect the increase in the transmitter content of individual vesicles, provided that the postsynaptic sites were not saturated. Since it has previously been shown that the infusion of a high concentration of L-glutamate into mature calyceal terminals did not cause saturation of AMPA receptors [23], enhancement of the transmitter content of individual vesicles may have been caused by nerve ligation. Taken together, our results thus provide the evidence that excitatory synaptic transmission is subject to divergent plasticity in different peripheral nerve injury models.

The strength of a synaptic connection between two neurons reflects three quantal parameters of neurotransmission: the number of a pool of available quanta (*N*); the probability of transmitter release (*p*); and the amplitude of the response attributable to quantal release (*q*) [20]. Although we defined *N *as the number of available vesicles, it is generally believed that *N *corresponds to the number of release sites or active zones that contain clusters of vesicles, some of which are docked near the presynaptic membrane [24]. Thus, it is likely that the enhanced quantal content with inflammatory pain reflects an increase in the probability of release of available quanta, with perhaps also increases in the number of release sites. In other words, two types of changes can be conceivable in ACC synapses with peripheral inflammation: (1) increase in the probability of activating exocytosis of a docked vesicle or (2) increase in the probability that a release site is occupied by a docked vesicle ready for release [20]. It has been suggested that 'kiss-and-run' vesicle fusion exists at endocrine cells, pituitary nerve terminals, cultured hippocampal neurons, and calyx of Held synapses [25]. The kiss-and-run can rapidly recycle vesicles and affect the size and the kinetics of the synaptic current. Although little is known whether kiss-and-run plays a major mode of fusion in ACC synapses, such mode of fusion may also be altered with inflammatory pain and nerve ligation. Future studies are necessary to understand the relationship between the enhanced quantal content and 'kiss-and-run' vesicle fusion at ACC synapses with chronic pain.

### Calcium-stimulated adenylyl cyclase and possible presynaptic action

We have previously shown that adenylyl cyclase [5] 1 and 8 are essential for chronic pain. For example, in AC1/8 double knockout mice, chronic pain sensitization was significantly reduced in inflammatory [15] and neuropathic pain model [7,21]. In ACC synapses with inflammatory pain, inhibition of AC1 and/or AC8 were sufficient to inhibit the long-lasting enhanced presynaptic transmitter release [15]. Thus, the molecular mechanisms responsible for the enhancement of probability of transmitter release and number of available vesicles in inflammatory pain could be mediated by presynaptic AC1 and/or AC8 proteins such as cAMP response element binding protein (CREB) [21]. In addition, we have found that AC1 is critical for both long-term presynaptic and postsynaptic changes in ACC synapses after nerve ligation [7]. AC1 thus may contribute both presynaptically and postsynaptically to nerve ligation-induced plastic changes in the ACC. Therefore, AC1 may serve as a potential therapeutic target for treating chronic pain.

### Characteristics of mEPSCs in pain related brain regions

Results obtained from the analysis of mEPSCs reveal that the rate and the amplitude of mEPSCs in spinal cord are significantly lower than those in ACC and basolateral amygdala. Nociceptive transmission starts from the peripheral terminals of dorsal root ganglion (DRG) to DRG soma, then to spinal cord dorsal horn and finally reaches to supraspinal structures such as brainstem, thalamus and pain-related cortex including ACC and insular cortex. Considering the nociceptive transmission pathway, spinal cord plays essential roles as not only the first site of synaptic integration for pain transmission but also the site to convey pain transmission to the supraspinal structure. Therefore, the excitability of the spinal dorsal horn neurons may be maintained at a higher level than other pain-related brain areas even at resting condition. Why are the rate and amplitude of mEPSCs in ACC synapses significantly lower than those in basolateral amygdala and spinal cord? Although it remains unclear why ACC synapses display low-rate low-amplitude mEPSCs compared to basolateral amygdala and spinal cord, it is strongly suggested that glutamatergic input to ACC synapses is low at resting condition. Probably, the excitability of ACC synapses may be maintained at a lower level under the resting condition, because ACC has to be activated in case of placebo analgesia/opioid analgesia as well as pain perception, two opposite physiological processes [9]. Thus, a systemic analysis of mEPSCs can provide us detailed information on synaptic transmission under physiological and pathological pain conditions. Future studies on quantal analysis in other pain-related brain regions with inflammatory and neuropathic pain will expand our knowledge to understand the synaptic mechanisms underlying chronic pain.

In summary, we demonstrate the strong evidences that the enhancement of both the probability of transmitter release and number of available vesicles contributes to the enhanced synaptic efficacy in ACC synapses with inflammatory pain, whereas only the enhancement of the probability of transmitter release underlies the enhanced excitatory synaptic transmission in ACC synapses with nerve ligation. Thus, peripheral nerve injury models are not all equivalent with respect to the excitatory synaptic transmission in ACC synapses. The mechanisms underlying these differences provide critical synaptic insights into physiological and pathological response to chronic pain and need to be clarified to alleviate chronic pain.

## Materials and methods

### Animals

All mice were maintained on a 12 h light/dark cycle with food and water provided *ad libitum*. The Animal Care and Use Committee of the University of Toronto approved all experimental procedures. Adult (6–8 weeks) male mice were used in this study.

### Inflammatory pain and neuropathic pain models

To induce inflammatory pain, 10 μl of 50% CFA (Sigma, St. Louis, MO) was injected subcutaneously into the dorsal surface of one hindpaw [14,15]. As a sham group, saline was injected. A model of neuropathic pain was induced by the ligation of the common peroneal nerve (CPN) as described previously [26]. Briefly, mice were anesthetized by intraperitoneal injection of a mixture saline of ketamine and xylazine. The left CPN was ligated with chromic gut suture 5-0 (Ethicon) slowly until contraction of the dorsiflexors of the foot was visible as twitching of the digits. The mechanical allodynia was tested on postsurgical day 7, and the mice were used for electrophysiological studies on postsurgical days 7–14. Sham surgery was done on the left leg by incising the skin, subcutaneous fascia, and the intercompartmental fascia. The muscle was then pulled laterally and posteriorly to expose the nerve. Thereafter, the skin was sutured with 5-0 silk suture.

For the measurement of behavioral responses, mice were placed in a round container and allowed to acclimate for 30 min before testing. Mechanical allodynia was assessed based on the responsiveness of the hindpaw to the application of von Frey filaments (Stoelting) to the point of bending. Positive responses include licking, biting, and sudden withdrawal of the hindpaw. Experiments were performed to characterize the threshold stimulus. Mechanical pressure from a 1.65 filament (force, 0.008 g) was found to be innocuous in normal mice. This filament was then used to test the mechanical allodynia after CFA injection and nerve ligation. Mechanical allodynia was tested five times with an intertrial interval of 10 min. Animals were then permitted a rest period for 20 min, after which mechanical allodynia was again tested. After obtaining the positive responses, the mice were used for electrophysiological recordings.

### Slice preparation

Coronal brain slices (300 μm) containing the ACC, basolateral amygdala, and spinal cord were prepared as described previously [15,27,28]. Slices were transferred to submerged recovery chamber with oxygenated (95% O_2 _and 5% CO_2_) artificial CSF containing the following (in mM): 124 NaCl, 2.5 KCl, 2 CaCl_2_, 2 MgSO_4_, 25 NaHCO_3_, 1 NaH_2_PO_4_, and 10 glucose at room temperature for at least 1 h.

### Whole-cell patch-clamp recordings

Whole-cell patch-clamp recordings were performed in a recording chamber on the stage of an Axioskop 2FS microscope (Zeiss, Oberkochen, Germany) with infrared differential interference contrast optics for visualization of whole-cell patch-clamp recording. Evoked EPSCs (eEPSCs) were recorded from layer II/III neurons with an Axopatch 200 B amplifier (Molecular Devices, CA), and the stimulations were delivered by a bipolar tungsten stimulating electrode placed in layer V of the ACC. Unitary EPSCs (uEPSCs) were obtained by repetitive stimulations for 100 trials at 0.1 Hz, and neurons were voltage clamped at -70 mV. The stimulus threshold was determined by applying a series of stimuli with increasing intensities until detectable currents were obtained, and the stimulus intensity (5 V) just above the level that gave mostly failures was used in the present study. Average amplitude and coefficient of variation (CV) of uEPSCs were calculated from 100 consecutive sweeps. The recording pipettes (3–5 MΩ) were filled with solution containing the following (in mM): 145 K-gluconate, 5 NaCl, 1 MgCl_2_, 0.2 EGTA, 10 HEPES, 2 Mg-ATP, and 0.1 Na_3_-GTP, adjusted to pH 7.2 with KOH. For mEPSC recording in the ACC, 1 μM TTX was added in the perfusion solution following eEPSCs recordings. For mEPSC recording in the ACC, basolateral amygdala, and spinal cord, 1 μM TTX was added in the perfusion solution. Picrotoxin (100 μM) was always present to block GABA_A _receptor-mediated inhibitory synaptic currents in all experiments. Access resistance was 15–30 MΩ and monitored throughout the experiment. Data were discarded if access resistance changed >15% during an experiment. Results were expressed as mean ± SE except the amplitude of uEPSCs (mean ± SD). Rise time and decay time were defined as reported previously [15]. Statistical comparisons were performed using *t*-test or ANOVA followed by Fisher's PLSD (protected least significant difference) post-hoc test. In all cases, *p *< 0.05 was considered statistically significant. Since no differences were found between saline-injected and sham operated groups, these data were pooled in a single control group.

## Competing interests

The authors declare that they have no competing interests.

## Authors' contributions

HT, MGZ and MZ participated in the conception, design, and interpretation of the study. HT and MGZ carried out the experiments, HT wrote the manuscript.
